# A retrospective study of smart spot, a Bluetooth low‑energy real‑time location system supporting quality improvement efforts in Uganda

**DOI:** 10.1371/journal.pdig.0001790

**Published:** 2026-11-03

**Authors:** Daniel Choi, Ediketh Tugume, Isaac Mwesigye, Aine Ivan Aye Ishebukara, Charly Huxford, Yashodani Pillay, Gotharido Tumubugane, Nathan Kenya-Mugisha, Ronald Kasyaba, J. Mark Ansermino, Dustin Dunsmuir, Pediatric Sepsis CoLab

**Affiliations:** 1 Department of Anesthesiology, Pharmacology and Therapeutics, University of British Columbia, Vancouver, British Columbia, Canada; 2 Institute for Global Health, BC Children’s Hospital and BC Women’s Hospital + Health Centre, Vancouver, British Columbia, Canada; 3 Uganda Martyrs’ Ibanda Hospital, Ibanda, Uganda; 4 World Alliance for Lung and Intensive Care Medicine in Uganda, Kampala, Uganda; 5 Uganda Catholic Medical Bureau, Kampala, Uganda; Iran University of Medical Sciences, IRAN, ISLAMIC REPUBLIC OF

## Abstract

Real-time location systems track assets and individuals, and in high-income clinical settings, they have improved patient flow. However, their use in low- and middle-income countries remains limited. Smart Spot, a low-cost Bluetooth low-energy real-time location system was integrated within the Smart Triage quality improvement platform in the outpatient department of Uganda Martyrs’ Hospital Ibanda. The primary objective was to evaluate whether Smart Spot data could be used to detect differences in time spent across clinical locations associated with Smart Triage implementation. The secondary objective was to assess the accuracy of Smart Spot data. At triage, children (or their caregivers) received a beacon attached to a lanyard. The beacons tracked patients’ locations throughout their time in the outpatient department. Smart Spot data were collected during a 4‑month baseline phase (pre-Smart Triage) and a 12‑month Smart Triage intervention phase. An algorithm was developed to reduce noise and generate more accurate records of each patient’s journey. The resulting pathways were compared to expected paths to evaluate Smart Spot’s accuracy. The median durations of patient time spent in specific areas were compared between phases using quantile regression. Data cleaning improved the matching to the expected patient flow from 13.0% to 73.1%. Compared with baseline, the median time patients spent in the assessment area decreased by 51.7% (-81.9 min; CI: -95.1 to -68.8), in the emergency room by 69.1% (-44.3 min; CI: -67.7 to -20.8), and total visit duration by 58.2% (-122.9 min; CI: -138.4 to -107.3), all with p-value < 0.001. Real-time location data supported the demonstration of the Smart Triage platform’s benefits. In the cleaned dataset, the intervention phase had shorter total visit time and time reductions within specific areas. Our evaluation demonstrates Smart Spot’s feasibility as an effective data-collection platform for quality improvement and its utility for retrospective analyses in low-resource settings.

## Author summary

Healthcare infrastructure in low- and middle-income countries often falls behind that of high-income countries. Significant resource constraints in these settings contribute to this difference, ultimately resulting in patients lacking the optimal care they need. Additionally, without digital health infrastructure tools, low-resource healthcare settings do not have the resources to record any data to track their progress on quality improvement goals. To address this issue, we developed Smart Triage, a triage platform for children in the outpatient department of hospitals in Uganda. The platform provides data-driven triage and can also be used to track quality improvement. Our study focused on Smart Spot (a wireless tag), which is part of Smart Triage and offers live tracking of patients’ locations. We found that the data collected through Smart Spot was noisy, so we developed a method to clean this data to provide a more accurate measurement of the children’s movement. While the system can benefit from further accuracy improvements, it showed us where improvements were made during the Smart Triage implementation. Our study shows that an affordable tracking approach can be used to monitor the process of care, guide local improvements, and enhance patient outcomes.

## Introduction

Digitalization has fundamentally reshaped clinical and industrial ecosystems by enabling real-time, data-driven decision-making. In healthcare, the rapid adoption of electronic health records (EHRs) and telemedicine has elevated expectations for seamless information exchange and patient-centred care [1]. Organizations now rely on integrated information systems that can share accurate, high-frequency data to optimize safety, throughput, and resource utilization.

Real-time location systems (RTLS) can be a valuable data source within a digital system. RTLS platforms can generate spatial-temporal data streams that locate items through wireless communication, such as Bluetooth Low Energy (BLE), passive or active radio-frequency identification (RFID), ultra-wideband (UWB), Wi-Fi, or emergent 5G [2]. BLE solutions offer low power consumption and room-level accuracy, whereas UWB or hybrid RFID-UWB architectures can achieve sub-meter precision at a higher cost and deployment complexity [3,4]. These technological trade-offs enable teams to tailor RTLS architectures to context-specific constraints in terms of accuracy, range, and budget. In healthcare, RTLS have demonstrated benefits in numerous studies, including cost reduction, improvements in care quality and work processes, and increased patient satisfaction [5]. These developments reflect a broader shift toward the Hospital 4.0/5.0 paradigm, in which smart sensors enable real-time data collection to optimize resource management and support data-driven patient care [6].

A systematic review of 42 peer-reviewed manuscripts focused on healthcare delivery concluded that RTLS deployments consistently enhanced patient safety, asset utilization, and workflow transparency [7]. All 42 studies identified by Overmann et al. were conducted in higher-resourced health systems in North America, Europe, and Asia, with a notable gap in low-resource settings. Consequently, current literature offers limited insight into RTLS deployments in resource-constrained environments and provides insufficient evidence to determine whether the substantial benefits reported elsewhere can be seen where infrastructure, staffing, and capital budgets are most constrained.

This study evaluates Smart Spot functionality, an RTLS implemented in Uganda. Smart Spot is an optional component of Smart Triage, a digital-health platform designed to streamline pediatric triage and clinical workflows and reduce patient wait times among critically ill patients in low-resource settings [8]. We describe the system’s implementation and performance, with a primary objective of assessing patient flow changes associated with Smart Triage implementation using Smart Spot as the measurement platform. The secondary objective was to evaluate the accuracy and feasibility of Smart Spot as a real-time location system in a low-resource setting. Overall, we aim to investigate how the automated location data of Smart Spot can advance Quality Improvement (QI) initiatives.

By systematically assessing RTLS deployment in a Ugandan hospital, this study adds much-needed evidence from a low-resource health system, a context largely missing from the current literature.

## Methods

### Ethics

Ethics approval for this study was obtained from the institutional review boards at Makerere University School of Public Health in Uganda (SPH-2021-41) and the Uganda National Council for Science and Technology (HS1745ES). As this is a QI project, patient consent was waived. Ethics approval for this study was not required by the University of British Columbia’s (UBC) Research Ethics Board.

### Study setting

The Smart Spot system was implemented in the outpatient department (OPD) of Uganda Martyrs’ Hospital - Ibanda (UMHI), Uganda. UMHI is a non-profit 178-bed hospital affiliated with the Uganda Catholic Medical Bureau, which admits about 1900 children (less than 12 years old) a year and serves a predominantly rural population. The emergency room is open at all hours, with the OPD functioning from 8:00 to 17:00 each day. This intervention was evaluated as a secondary analysis of the Smart Triage study. The Smart Spot hardware was installed and piloted for 1.5 months prior to February 2022. Data collection then proceeded in two phases: a four-month baseline period from February 2022 to May 2022, during which routine clinical practice remained unchanged but patient movements and treatments were tracked; and a twelve-month intervention period from June 2022 to May 2023, during which Smart Spot operated continuously as part of the Smart Triage platform.

During both the baseline and intervention phases, the data-collection procedures remained unchanged. The only substantive change between phases was the implementation of the Smart Triage quality-improvement platform. Smart Spot data were collected continuously across both phases and used solely for retrospective evaluation of patient flow.

### Hardware

The hardware architecture was chosen to minimize costs and maintenance requirements for a low-resource setting. The components of the hardware architecture were:

1. Portable BLE beacons. Keychain - K11 [9]
2. Bluetooth Gateways - G1 IoT [10]
3. A WiFi enabled server running Linux Ubuntu [11]

S1 Table and S2 Table provide full manufacturer details.

After triage, a BLE beacon attached to a lanyard was given to the child or caregiver to wear during their time in the OPD. During the intervention period, the lanyards were colour-coded with the triage category. Emergency patients (red lanyards) or priority patients (yellow lanyards) could be quickly identified among all the non-urgent patients (green lanyards). The beacon and lanyards, which were all one colour during baseline, were retrieved from the patient when they were leaving the OPD. Additional beacons in each treatment room represented specific interventions such as intravenous antibiotics or supplemental oxygen. Each of the beacons had a button that was used for recording treatments. Gateway devices were mounted on the walls in clinical locations, including the waiting room, consultation rooms, injection room, laboratory, and emergency room (Fig 1). A server box situated in the outpatient department processed data sent from the gateways and then updated the clinician dashboard website available on the local WiFi network.

**Fig 1:**
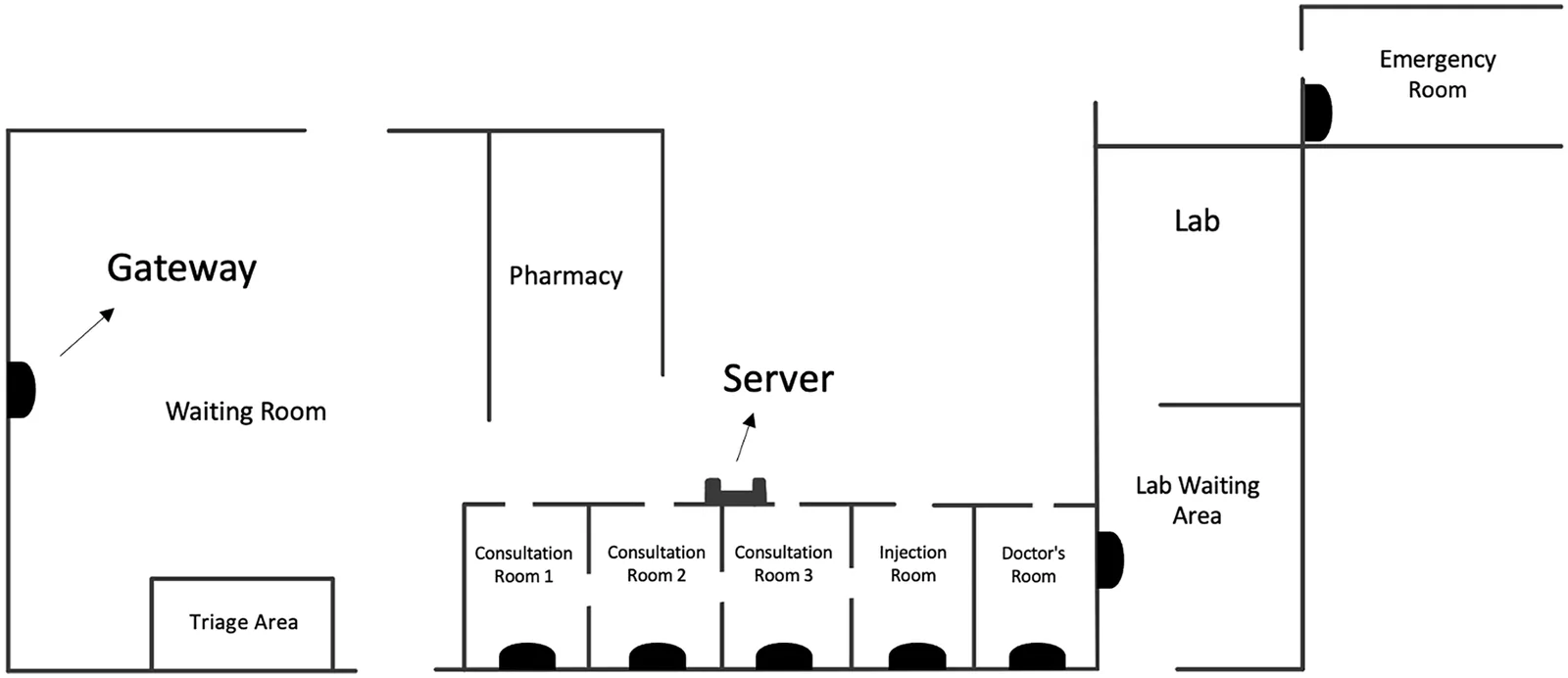
The layout of the outpatient department at Uganda Martyr’s Hospital Ibanda (not to scale).

### Software

Gateways were configured with a manufacturer-specific Media Access Control (MAC) filter so that only Smart Spot beacons were detected. Once per second, each gateway transmitted data to the local server that included the MAC address of every beacon detected, and its corresponding received signal strength indicator (RSSI) measured in decibels (dB). An RSSI value is the measurement of the strength of a wireless signal received by the gateway [12] and they were used to determine patient locations. Each gateway (room) had an RSSI threshold that represented a room-specific reference signal strength roughly equivalent to the signal strength of a beacon in the room entrance. If a gateway read a stronger signal than this reference, the beacon was likely closer to the gateway than the entrance, and thus in the room. Room-specific thresholds were empirically determined using a trial-based calibration procedure (S1 Fig). For each room, a beacon was placed inside the room, and the threshold was set 5 dB above the weakest in-room smoothed RSSI observed. The beacon was then placed just outside the room to confirm that outside-room RSSI values fell at least 5 dB below the threshold. This in/out procedure was repeated across multiple trials, and thresholds were retained when they consistently produced correct in-room/out-of-room status updates for at least 9 trials.

A smoothed measurement of the beacon to gateway signal strength at 1 Hz was computed as the 75th percentile of the 20 most recent RSSI values (the last 20 seconds) for the gateway-beacon pair. The location-determining algorithm (Fig 2) was then run once per gateway-beacon pair (representing a room-patient pair). A patient’s status was always a room (Waiting Room, Consultation Room, etc.) or “Travelling” for a patient not currently in any of the included rooms. The algorithm used the smoothed RSSI for this gateway-beacon pair (denoted RSSI_A_ for room A), the room A RSSI threshold, and the patient’s starting status (in this room already, in another room, or “Travelling”), and their time spent so far at this starting location. There were 3 possible outcomes of the algorithm:

**Fig 2:**
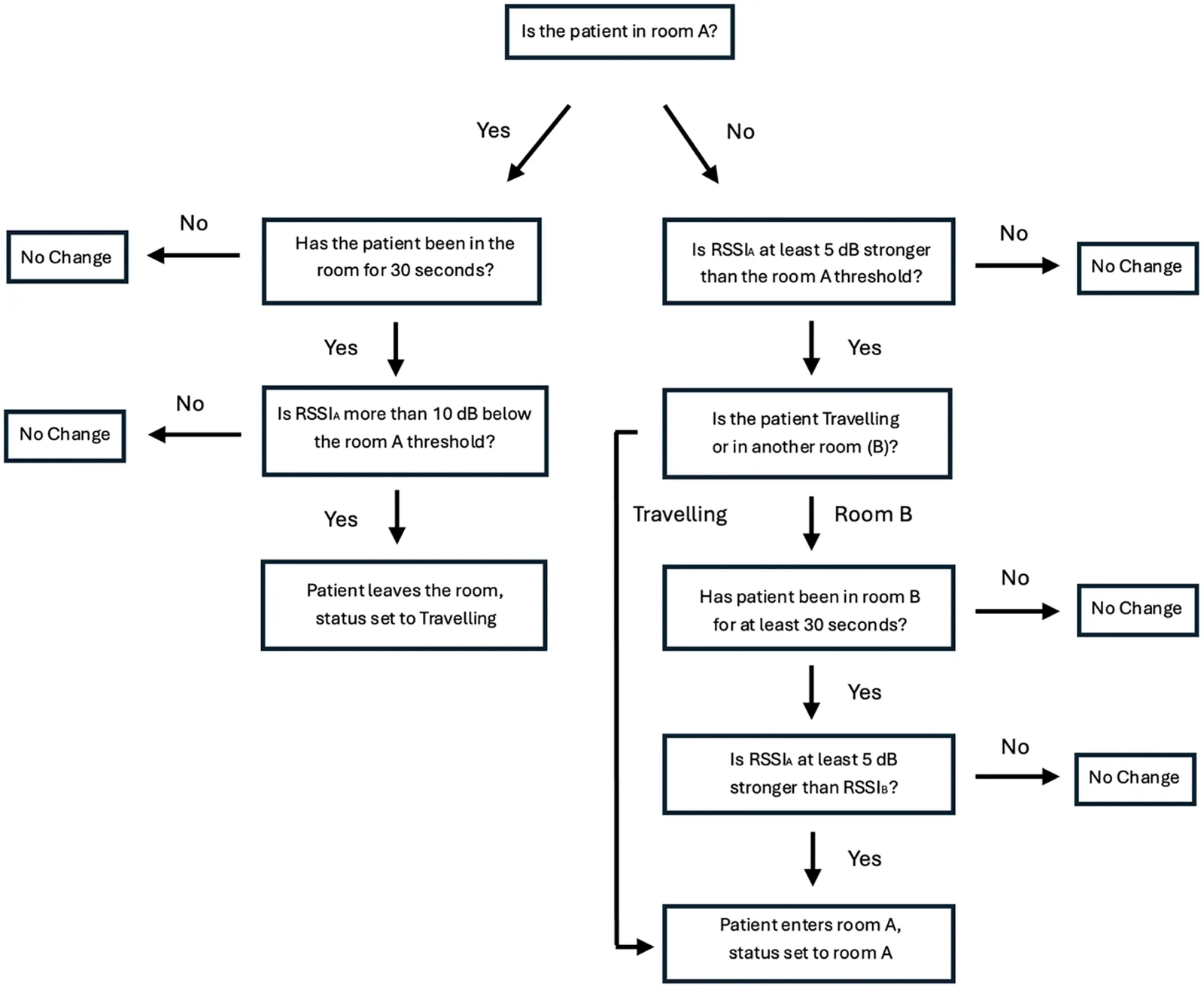
A flow chart showing the location-determining algorithm. Received Signal Strength Indicator (RSSI). The subscript indicates the room (RSSI_A_).

1) The patient exited room A, with their status changed to “Travelling”.
2) The patient entered room A, with the status changed to room A.
3) No change was made to the patient’s status.

If the patient was in room A for at least 30 seconds and then the RSSI_A_ was more than 10 dB below the threshold the patient was set to Travelling (1). If on the other hand RSSI_A_ was at least 5 dB stronger than the room threshold and they were Travelling or in a different room B for 30 seconds or more and RSSI_A_ was also at least 5 dB stronger than RSSI_B_ (their strength with the other gateway) then their status was updated to be in room A (2). If neither of the transition conditions were met, the patient’s location assignment remained unchanged (3).

Additionally, the software detected the coordinated tapping of patient and treatment beacon buttons to log the patients’ emergency treatments. Treatments were detected when a flag was received from tapping the buttons of both a treatment beacon and a patient beacon in the same location. The algorithm ignored a second tap that occurred within 20 seconds to avoid double-counting. The system logged a treatment event when it found the treatment flags sent from both patient and treatment beacons in the same location within a 10-second time frame. Overall, the Smart Spot system detected patient room changes and treatment events in real-time, and the Smart Triage clinician dashboard updated every five seconds to provide all OPD clinical staff with the patient information. The patient’s location is listed as either a specific location or as “Travelling”, when the patient is not currently located in any of the included rooms.

### Data cleaning and analysis

Smart Spot data, stored on the local server at UMHI, were automatically de-identified and sent to a secure server at the University of British Columbia. Smart Spot study data are now available through the Pediatric Sepsis Data CoLaboratory [13], a global network for collaboration and data sharing to address pediatric sepsis morbidity and mortality [14]. The data generated by the Smart Spot system contained noise due to signal interference in the RSSI values between adjacent rooms. An algorithm was developed using RStudio (Version 2024.12.1 + 563) and R (Version 4.4.2, 2024-10-31) retrospectively for this analysis. This was used to clean the noise and analyze the data from the baseline and intervention phases. This algorithm removed any room logs shorter than 60 seconds or following a ≥ 12‑hour gap, and grouped adjacent rooms (waiting, consultation, injection and doctor’s room) under the category of “assessment area”. The 60-second cutoff was selected to remove brief room detections that were unlikely to represent true patient presence, as room logs shorter than 60 seconds occurred frequently and did not realistically reflect actual movement between clinical locations. The ≥ 12-hour gap threshold was chosen to remove logs that may have been created due to the beacon remaining active after patient discharge or admission and then appearing again the next day still tied to the same patient, which is not realistic in the OPD. These thresholds were selected based on inspection of the raw data distributions and alignment with typical outpatient workflows at the study site.

Once cleaned, the accuracy of this algorithm was validated by checking each recorded patient path against the standard clinical pathways, which were defined through consultation with on-site clinical staff familiar with workflows. Specifically, the cleaned patient trajectories were compared against these staff-defined pathways to assess alignment and algorithm accuracy. The patient pathways used can be found in S2 Fig. Median patient durations were then computed for four locations, total OPD stay, assessment area, emergency room, and laboratory, for both the baseline and intervention periods. Differences between phases were calculated, and the statistical significance of this was tested with quantile regression, which focuses on the median and is less sensitive to skewed distributions. There were no additional covariate adjustments beyond the study phase.

Box-and-whisker plots generated in R provided a clear visual summary of the results. A pseudocode description of the data-cleaning algorithm is shown in S3 Fig and the full implementation code and documentation is available in the Pediatric Sepsis Data CoLaboratory’s Borealis repository [15].

## Results

Before signal cleaning, only 13.0% of pathways aligned with the expected pathways; this proportion increased to 73.1% after application of the Smart Spot data-cleaning algorithm. Compared to baseline, the Smart Triage intervention period had significant reductions in median time spent in the assessment area (-81.9 minutes [-51.7%]; 95% Confidence Interval (CI)= -95.1 to -68.8), emergency room (-44.3 minutes [-69.1%]; CI = -67.7 to -20.8), and total visit duration (-122.9 minutes [-58.2%]; CI = -138.4 to -107.3), all at least p < 0.001. The 0.6-minute increase in median laboratory time was not statistically significant (p = 0.57) (Table 1, Fig 3).

**Table 1:** Time spent in each area of the outpatient department during the baseline and intervention phases.

Event	Median Time in Baseline (minutes)	Median Time in Intervention (minutes)	Change in Median Time (minutes)	p value	95% Confidence Interval (minutes)
Total	211.0	88.1	−122.9	<0.0001	−138.4 to −107.3
Assessment Area	158.5	76.6	−81.9	<0.0001	−95.1 to −68.8
Emergency Room	64.0	19.7	−44.3	<0.001	−67.7 to −20.8
Lab	17.4	18.0	0.6	0.57	−1.5 to 2.7

**Fig 3:**
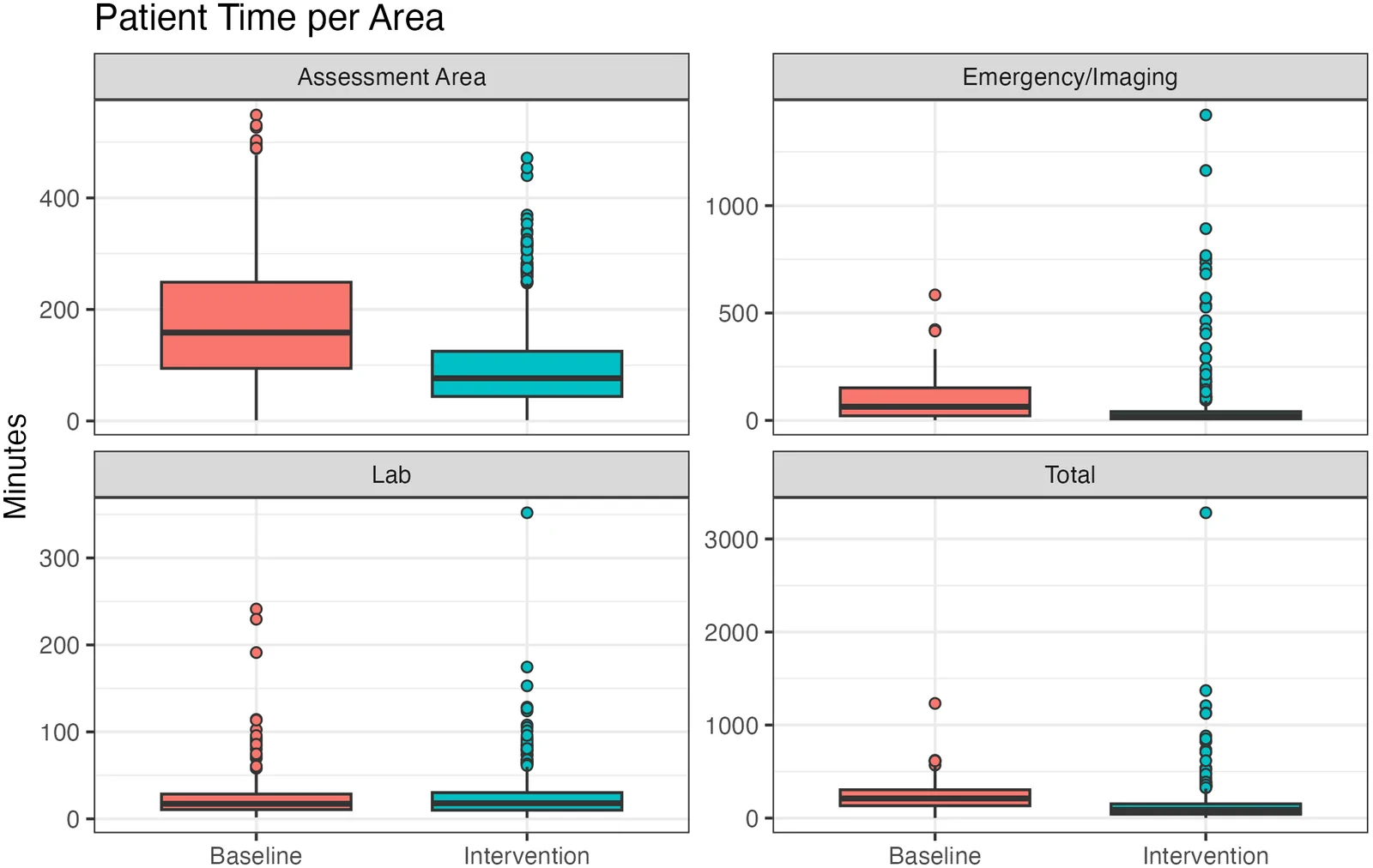
A box plot of the median time the patient spent in each area of the outpatient department in the baseline and intervention phases.

## Discussion

Smart Spot, a low cost BLE RTLS, provides real time patient information to staff and retrospective aggregated data to support QI. During the study we found that Smart Spot requires data cleaning for the data to generate useful outputs. The raw data had a low percentage of matches with expected clinical pathways, demonstrating the need for enhanced data cleaning for accurate live patient tracking. However, with the cleaning algorithm, Smart Spot can be used as a QI tool to identify workflow bottlenecks and guide system improvements. For example, location data can identify areas where patient wait times are longest, such as at the laboratory, prompting changes to reduce delays. Tracking the timeliness of emergency treatments can reveal inefficiencies in service delivery and inform interventions to improve care. By providing objective, continuous data, Smart Spot enables teams to move beyond anecdotal observations and guide QI efforts with objective evidence.

Collecting health information system data in low- and middle-income countries is limited by low staff motivation, reluctance to adopt new tools, absent or unclear implementation protocols, variable leadership, and shortages of technical personnel [16]. These factors make manual entry, whether on paper or into electronic systems, highly dependent on individual skill and sustained supervision, which can result in inconsistent and incomplete records. Smart Spot mitigates these constraints by automating a system composed of beacons, gateways, and a local server. The entire process of data collection and processing minimizes the overall human input required. This standardized system reduces training and time demands, minimizes human errors, and is less sensitive to turnover or variable staff capabilities than manual entry into EHRs.

### Rationale for BLE-based Real-time location systems

Smart Spot leverages BLE technology composed of inexpensive, low-power beacons and gateways [17]. Other RTLS solutions, including UWB (Ultrawideband) are capable of centimetre-level accuracy but have high installation costs [18]. RFID (Radio Frequency Identification) is similarly capable of centimetre-level localization accuracy when equipped with Ultra-High Frequency (UHF) [18]. However, UHF RFID requires the gateways to be deployed with a density of 1 gateway for every square metre, making it impractical and costly [16]. By contrast, the implementation and operation costs of BLE are relatively lower [18], and a previous analysis demonstrated the cost-effectiveness of the Smart Triage QI platform [17]. Given the cost, energy, and maintenance constraints in low-resource clinics, BLE is a practical choice for Smart Spot.

### Deployment and scalability considerations

The Smart Spot system was designed to be easily deployable in other low-resource hospital settings. As with Smart Triage, it is important to get approval from the hospital leadership and appropriate health authorities and establish local champions within the healthcare team for the success of Smart Spot at a new site. These human issues would likely require far more effort than the technical deployment. Deployment requires minimal, low-cost hardware and software. Installation does not require continuous or extensive modifications, and system configuration can be completed using existing clinical room layouts by following the RSSI threshold choosing algorithm (S1 Fig). Smart Spot may be adaptable to other outpatient departments with resource constraints.

If an EHR is already being used in the OPD at a new site, then Smart Spot should be used to provide a patient location to the existing EHR, rather than as a separate stand-alone system. This will minimize the staff training and IT support needs. Using the Smart Spot BLE hardware with the Smart Spot location algorithm (Fig 2) developed in the EHR, once the RSSI threshold choosing algorithm (S1 Fig) is setup for each gateway, the EHR can collect useful patient location data for quality improvement efforts.

### Security and privacy considerations

The use of Internet of Things (IoT) technologies for real-time health data collection introduces security and privacy considerations. IoT-enabled systems often rely on wireless communication, distributed sensing devices, and centralized data aggregation, which can introduce vulnerabilities such as unauthorized device access or interception of transmitted data. These challenges may be amplified in low-resource settings, where limitations in cybersecurity infrastructure can increase the risk of data misuse.

In this study, privacy risks were limited, as data sent by the BLE hardware to the server contained only signal strength values, not any patient health data. Devices are only referred to by MAC addresses which are mapped to individual beacons only within an internal table on the Smart Triage server and these beacons are reused often. The server then is the one piece of Smart Spot hardware with sensitive data that needs to be protected. At Ibanda hospital, the server is located in a locked cage, high out of reach in the high traffic assessment area and this area is locked outside OPD hours. Smart Triage data is accessible only to those who have a device that is connected to the WiFi network and also have an account to login to the Smart Triage dashboard. Regular users can only see patients that have visited the hospital in the last 24 hours, while admin users can see long term reports, but they show only aggregate numbers, no individual patient data. The use of Smart Spot with Smart Triage in no way decreases the security of the Smart Triage data, nor exposes any healthcare data.

### Limitations

The main limitation of the Smart Spot data was residual noise, with frequent bouncing between patient locations, particularly in adjacent rooms within the OPD (Fig 1). Data cleaning improved the quality, though some noise persisted. While cost and sustainability are critical considerations in choosing an RTLS for low-resource settings, the residual noise reflects the trade-off of using a low-cost system.

A key methodological limitation of this study is its before-after design, which lacks a concurrent control group. Without a comparison group observed over the same period, it is not possible to rule out that temporal trends, seasonal variation, or other concurrent changes such as staff levels or patient demographics at the facility contributed to the observed improvements. This limits causal inference and means the findings should be interpreted as associations rather than evidence of a direct effect of the Smart Triage intervention.

Furthermore, there is an imbalance between study phases. The baseline period was substantially shorter than the intervention period (4 months vs. 12 months) and included fewer patients (589 vs. 1319 after cleaning). This imbalance may introduce bias due to unmeasured temporal or seasonal factors. Additionally, there may have been changes in staffing levels or patient mix between the baseline and intervention periods that were independent from the introduction of Smart Triage. As a result, the observed 58.2% reduction in total visit duration should be interpreted cautiously, as the contribution of staffing or other operational changes independent of Smart Triage cannot be quantified from the available data. Quantile regression reduced this risk of distortion from outliers by focusing on medians, but some observed differences may partly reflect influence by confounding factors rather than the intervention alone.

In addition, location resolution was limited after cleaning because several rooms were merged into a single “assessment area”. This step was necessary to align location logs with expected patient flow and to generate valid time-in-area estimates. However, aggregation reduced the ability to distinguish specific service points (such as time in the waiting room vs. time with a clinician). As a result, delays occurring within individual rooms could not be independently resolved, limiting the ability to identify room-specific workflow bottlenecks. Future work could focus on improving resolution of outpatient rooms (e.g., optimizing beacon placement, potentially with multiple beacons per room, refining RSSI room thresholds). Currently, each room contains a single gateway. Because adjacent rooms are in close proximity, their RSSI signals can overlap, making it difficult to accurately determine which room a patient is in. This signal noise was the primary reason several rooms were merged into a single area during cleaning. Installing additional gateways within individual rooms would allow the system to compare signal strength readings from multiple positions. A patient physically inside a room would be expected to produce a strong signal to all gateways within that room, whereas a patient in an adjacent room would not. This would provide a more accurate basis for room-level assignment. This could enable more reliable differentiation between adjacent rooms and thereby support more targeted quality improvement initiatives.

Cost and resource constraints are important considerations for implementation. Our team introduced rechargeable beacons after the study to improve cost-effectiveness by reducing expenses associated with battery replacements. The risk of loss is another potential limitation of reusable beacons, which we mitigated through the implementation of appropriate training programs and the use of a beacon detector with an audible alarm at the facility gate. Due to the strong vigilance of the staff at UMHI, no beacons were lost during this study period.

Overall, the residual pathway mismatches remaining after data cleaning, the imbalance in duration and sample size between the baseline and intervention periods, and the potential for temporal or seasonal confounding constrain the strength of the conclusions that can be drawn from a single-site before-after design.

### Strengths

A key strength was the duration and scale of the data collection. Data were collected continuously for 16 months, capturing routine fluctuations rather than a short pilot snapshot. Additionally, the Smart Spot system reduced the burden on health workers by minimizing the time needed to record treatment times and automatically recording patient spatial-temporal data. Tapping beacons was quicker than manual entry, and treatment times were logged as care was delivered rather than entered retrospectively. Nearly 2000 pediatric visits were recorded, supplying sufficient size for statistical testing and allowing results to reflect day-to-day practice.

## Conclusion

Smart Spot enabled objective evaluation of workflow changes associated with the Smart Triage quality-improvement platform, demonstrating its feasibility for supporting retrospective quality-improvement analyses in low-resource settings. The system revealed statistically significant reductions in duration across all clinical areas except the laboratory*.* However, these findings should be interpreted cautiously, as the before-after study design and the absence of adjustment for potential confounding factors mean that the observed reductions cannot be solely attributed to the Smart Triage intervention. Despite the limitations associated with residual noise, the study demonstrated that affordable RTLS yield meaningful insights into workflow efficiency. Further implementations will aim to continuously improve the Smart Spot system. The current data cleaning algorithm supports ongoing QI objectives by enabling the generation of monthly reports on patient time spent in OPD areas.

## Supporting information

S1 Table Manufacturer details for K11 keychain portable Bluetooth Beacon. (DOCX)

S2 Table Manufacturer details for G1 IoT Bluetooth Gateway. (DOCX)

S1 Fig The full setup process for choosing an RSSI threshold for a gateway based on moving 10 BLE beacons into and out of the room with the goal of choosing the threshold that allows Smart Spot to accurately indicate when a beacon has entered and exited a room. (TIFF)

S2 Fig Standard clinical pathways at Ibanda Hospital. (DOCX)

S3 Fig The pseudocode for the data-cleaning algorithm applied to clean noise in Smart Spot data. (DOCX)
