# Supplementary material for: A retrospective study of smart spot, a Bluetooth low‑energy real‑time location system supporting quality improvement efforts in Uganda
Source: PLOS Digit Health. 2026 Nov 3;5(11):e0001790. doi: 10.1371/journal.pdig.0001790 (PMC13730383; doi:10.1371/journal.pdig.0001790)
Supplement: Supplementary file 1 [file pdig.0001790.s001.docx]

**S1 Table. Manufacturer Details for K11 Keychain Portable Bluetooth Beacon**

| Material for shell | ABS |
| --- | --- |
| Color | Black, white |
| Size | 36*36*5.6mm |
| Weight | 10g (with battery) |
| Battery type | CR2032 |
| Battery life | 14 Months |
