# Supplementary material for: A retrospective study of smart spot, a Bluetooth low‑energy real‑time location system supporting quality improvement efforts in Uganda
Source: PLOS Digit Health. 2026 Nov 3;5(11):e0001790. doi: 10.1371/journal.pdig.0001790 (PMC13730383; doi:10.1371/journal.pdig.0001790)
Supplement: Supplementary file 2 [file pdig.0001790.s002.docx]

**S2 Table. Manufacturer Details for G1 IoT Bluetooth Gateway**

| Weight | 155g |
| --- | --- |
| Power supply | DC 5.0 (+/- 5.0%) Max. 5.5 Volts |
| Wifi protocols | IEEE 802.11 b/g/n |
| Wifi frequencies | 2.4-2.4835 GHz |
