# Supplementary material for: A retrospective study of smart spot, a Bluetooth low‑energy real‑time location system supporting quality improvement efforts in Uganda
Source: PLOS Digit Health. 2026 Nov 3;5(11):e0001790. doi: 10.1371/journal.pdig.0001790 (PMC13730383; doi:10.1371/journal.pdig.0001790)
Supplement: Supplementary file 3 [file pdig.0001790.s004.docx]

Clinical pathway 1: Assessment Area

Clinical pathway 2: Assessment Area -> Emergency/Imaging Room

Clinical pathway 3: Assessment Area -> Lab -> Assessment Area

Clinical pathway 4: Assessment Area -> Lab -> Assessment Area -> Emergency/Imaging

Clinical pathway 5: Emergency/Imaging

Clinical pathway 6: Assessment Area -> Emergency/Imaging -> Assessment Area

Clinical pathway 7: Assessment Area -> Lab -> Assessment Area -> Emergency/Imaging -> Assessment Area
