# Supplementary material for: A retrospective study of smart spot, a Bluetooth low‑energy real‑time location system supporting quality improvement efforts in Uganda
Source: PLOS Digit Health. 2026 Nov 3;5(11):e0001790. doi: 10.1371/journal.pdig.0001790 (PMC13730383; doi:10.1371/journal.pdig.0001790)
Supplement: Supplementary file 4 [file pdig.0001790.s005.docx]

FOR each patient

SORT room logs by timestamp

CALCULATE room-stay durations:

FOR each consecutive pair of logs (log_i, log_{i+1})

duration_i = timestamp(log_{i+1}) - timestamp(log_i)

END FOR

REMOVE room logs where computed duration < 60 seconds

REMOVE room logs following gaps ≥ 12 hours

GROUP adjacent rooms (Waiting Room, Consultation Room, Doctors Room, Injection Room)

INTO "Assessment Area"

GENERATE cleaned patient pathway

END FOR

COMPARE cleaned patient pathways against standard clinical routes:

EXTRACT ordered room sequence from cleaned pathway

FOR each standard clinical route

IF cleaned sequence == standard route sequence

(same rooms visited in the same order)

MARK as a match

END IF

END FOR

CALCULATE median duration for:

- Total OPD stay

- Assessment Area

- Emergency Room

- Lab

COMPARE baseline vs intervention periods

TEST differences using quantile regression
